# Microplastics in irrigation water and vegetable garden soils adjacent to the Msimbazi river, Tanzania

**DOI:** 10.1007/s42452-025-07742-3

**Published:** 2025-09-30

**Authors:** James Joseph Mwesiga, Dativa Joseph Shilla, Daniel Abel Shilla

**Affiliations:** 1 Department of Aquatic Sciences and Fisheries Technology, University of Dar Es Salaam, P.O.Box 60091, Dar Es Salaam, Tanzania; 2 Department of Physical and Environmental Sciences, Faculty of Science, Technology and Environmental Studies, The Open University of Tanzania, P.O.Box 23409, Dar Es Salaam, Tanzania; 3 Department of Chemistry, Dar Es Salaam University College of Education, P.O.Box 2329, Dar Es Salaam, Tanzania

**Keywords:** Plastic pollution, Urban rivers, Irrigation water, Vegetable gardens, Plastic Carrier bags ban

## Abstract

Microplastics (MPs) are present in significant quantities across various environments; however, their persistence and detrimental effects on terrestrial and aquatic ecosystems remain poorly understood. This study has examined MPs in water from the Msimbazi River, used for irrigation, and from soils of nearby vegetable gardens. The results indicate a higher concentration of MPs downstream in the Msimbazi (14.33 ± 2.92 MPs per 10 mL of water) compared to upstream at Sukita (8.49 ± 2.47 MPs per 10 mL of water). A significant difference in MPs abundance was observed between the water samples collected from Sukita and Msimbazi sites (two-sample t-test, degrees of freedom (df) = 62, *P* < 0.001). Conversely, soil from Sukita gardens exhibited a lower abundance of MPs (28.00 ± 4.25 MPs per g of soil) compared to soils from Msimbazi gardens, which contained (34 ± 5.79 MPs per g of soil). Additionally, a significant difference in MPs concentration was found between soils from vegetable gardens in Sukita and Msimbazi (two-sample t-test, df = 62, *P* < 0.0001). Attenuated reflection transform infrared spectroscopy identified common plastic polymers from water and soil samples, including polyethylene terephthalate, low-density polyethylene (LDPE), polypropylene (PP), and polyesters. The results provide crucial insights into the abundance of LDPE (18.70–21.20%) and PP (20.50–22.10%) in the Msimbazi River water and soil of the adjacent vegetable gardens, respectively. These findings underscore the potential danger of MPs to the environment and the urgent need for better waste management strategies.

**Supplementary Information:**

The online version contains supplementary material available at 10.1007/s42452-025-07742-3.

## Article highlights

**Microplastics:**

First study to examine the co-occurrence of microplastics (MPs) in the water of the Msimbazi River and the soils of nearby vegetable gardens.

**Irrigation water:**

Primary source of microplastics (MPs) to the soil in vegetable gardens.

**Vegetable garden soils:**

A microplastics (MPs) hub from multiple pathways, containing a large amount of microplastics (MPs), up to 44,300/Kg.

**Plastic bags:**

The study has identified the predominant microplastic polymers linked to single-use plastic bags (LDPE) and reusable bags (PP) in both water and soil.

**Urban vegetable farming:**

The study highlights the vital role of the authorities in ensuring clean and safe spaces for urban vegetable farming.

**Supplementary Information:**

The online version contains supplementary material available at 10.1007/s42452-025-07742-3.

## Introduction

The global mass production of plastic materials, their easy availability, and poor waste management have raised significant concerns regarding their impacts on the environment and public health that necessitate immediate attention [15, 87]. Approximately 40% of plastic used recently is single-use plastic, which is often dumped illegally in soil, incinerated, or, most commonly, left in aquatic environments, including rivers [17, 72]. The degradation of plastic waste (macro plastics) into mesoplastics, microplastics (MPs), and nanoplastics (NPs) via abiotic (chemical and physical) and biotic (living) processes is a pressing concern [69]. Several factors, such as thermal, hydrology, and ultraviolet lights (photo degradation), facilitate abiotic degradation, while biotic degradation is influenced by the enzymatic reactions of microorganisms [49, 68]. MPs and NPs are defined as plastic particles smaller than 5 mm and less than 1 mm, respectively [52]. Nevertheless, poor plastic waste management, i.e., disposal along terrestrial and aquatic environments in developing countries, including most African countries, accelerates significant threats; thus, urgent actions are required [50, 73, 101].

Like many other urban municipalities in Africa and around the world, Tanzania faces challenges related to plastic pollution and its associated environmental and public health impacts [83]. For instance, Dar es Salaam, Tanzania, generates over 4,750 tonnes of solid waste daily, of which 10–16% consists of plastic waste [60]. Furthermore, more than 60% of solid waste, specifically single-use plastic, is unlawfully dumped by residents in river valleys [91]. In Tanzania, plastic waste is primarily classified as solid waste, and its management adheres to the Environmental Management Act (EMA) of 2004. This key legislation provides a framework for environmental management on Tanzania's mainland. It empowers Local Government Authorities (city authorities, municipal authorities, and Town Councils) to manage waste, promoting a local and practical approach. The Local Government Act of 1982 also enables municipal authorities to fully implement solid waste management practices according to their geographic jurisdictions [93]. Nevertheless, in 2019, the Tanzanian government enacted legislation banning single-use plastic carrier bags and encouraging the use of reusable bags [82]. Despite this, plastic bags, particularly single-use thin plastic bags, remain widely used. Additionally, as of 2022, Tanzania was still listed among the 20 African countries contributing significant amounts of plastic to the ocean through riverine discharge [31].

Similar to many other large urban rivers, the Msimbazi River in Dar es Salaam is polluted with various pollutants, including plastic waste, and discharges its contaminated water into the Indian Ocean [4, 113]. Several studies have reported the presence of plastic waste in the ocean, comprising macroplastics, MPs, and NPs, which have detrimental effects on marine ecosystems; these effects may extend to humans through the food chain [3, 11, 112]. Furthermore, unlike aquatic systems, terrestrial environments, including land, are particularly vulnerable to plastic pollution [44].

Sources of plastic in the terrestrial environment include domestic and industrial waste, landfills, soil mulching with plastic films, and atmospheric deposition [66]. Additionally, irrigation with water contaminated by plastics tends to introduce a significant quantity of MPs and NPs to farms, particularly vegetable gardens located near river shorelines [106, 116]. Primary MPs (from personal care products, cosmetics, and waste discharges from the plastic industry) and secondary MPs (resulting from the degradation of macroplastics) are commonly found in wastewater and terrestrial environments [70]. However, secondary MPs are more abundant than primary MPs [104, 150]. MPs in agricultural soil can negatively impact soil health by influencing its physical, chemical, and biological characteristics [21, 36, 39]. For instance, MPs such as polyethylene reduce soil water-holding capacity due to increased evaporation and temperature [121, 136]. Furthermore, MPs directly affect the biological properties of soil by altering the microbial community [76, 139] or indirectly by providing adsorption surfaces for pathogenic microorganisms, i.e., bacteria and viruses [53, 78, 86]. Also, MPs and NPs in agricultural soil can penetrate crops via seeds, leaves, or roots, and affect important aspects of plant physiology like growth and development, thus causing low crop yield or reduced crop biomass [8, 34, 42].

The additives and plasticisers incorporated during the industrial manufacture of plastic materials to enhance their physical stability are covalently bonded, yet they can be easily released into the environment, thereby posing a danger to public health [5]. For example, additives such as Bisphenol A and phthalates can cause cancer and endocrine disruption in humans [120]. Consequently, MPs, NPs, and their associated chemical additives in aquatic and terrestrial resources have created significant environmental and human health concerns [55, 96, 124]. Furthermore, MPs can adsorb environmental contaminants, including heavy metals, organic pollutants, and pathogenic microorganisms, for extended periods, transporting them over considerable distances due to their non-biodegradable and lipophilic nature [30, 64, 107]. Thus, MPs can carry and transmit pathogenic microorganisms and persistent organic and inorganic contaminants from polluted environments to humans. For instance, several pathogenic microorganisms, including *Salmonella sp,* have been isolated from plastic waste debris in aquatic environments, while *Escherichia coli* can remain on high-density polyethylene (HDPE) for at least 28 days while retaining its virulence [97, 98]. Therefore, it is essential to monitor contamination with plastics and MPs. The variations in surface structure and chemical composition of different plastic polymer types influence the microfauna and mesofauna that assemble on MPs [29, 138]. Soil micro and mesofauna ingest MPs and NPs into their food chain, impacting their growth and development in the terrestrial ecosystem [27, 131]. Regarding human health, exposure of children to MPs has been shown to cause adverse effects through cell damage leading to cancer [19, 141]. Studies have also reported health complications in humans, including obstruction of intestinal systems, blockage of enzyme production, and reduced growth rates resulting from the ingestion of MPs [113]. However, further research on the hazards and health effects of MPs on the human body is ongoing [110].

Despite the negative impacts of MPs as emerging contaminants on the environment and human health, Tanzania, like many African countries, lacks regulations or policies governing the concentration of MPs in irrigation water or agricultural soil [118]. Dar es Salaam, Tanzania's most populous city, relies heavily on urban agriculture to supply fresh vegetables. However, similar to many other urban areas in developing countries, Dar es Salaam's plans do not allocate sufficient land for vegetable cultivation due to space constraints. This results in most urban vegetable gardens being situated in polluted or uncontrolled environments, which poses significant challenges to their productivity and safety [119]. Unfortunately, there is insufficient information on the status of MPs’ pollution in urban river basins in Tanzania, including the Msimbazi River, whose water is frequently utilised for irrigation and from the soil of nearby vegetable gardens, particularly in the summer (June to October). Consequently, the objectives of this study were (1) To ascertain the quantity and types of MPs in water from the Msimbazi River and (2) To determine the quantity and types of MPs in the soils of adjacent vegetable gardens. The results can provide policymakers in Tanzania and other developing countries with an initial understanding of MPs as neglected environmental emerging pollutants.

## Materials and methods

### Description of the study sites

**Fig. 1:**
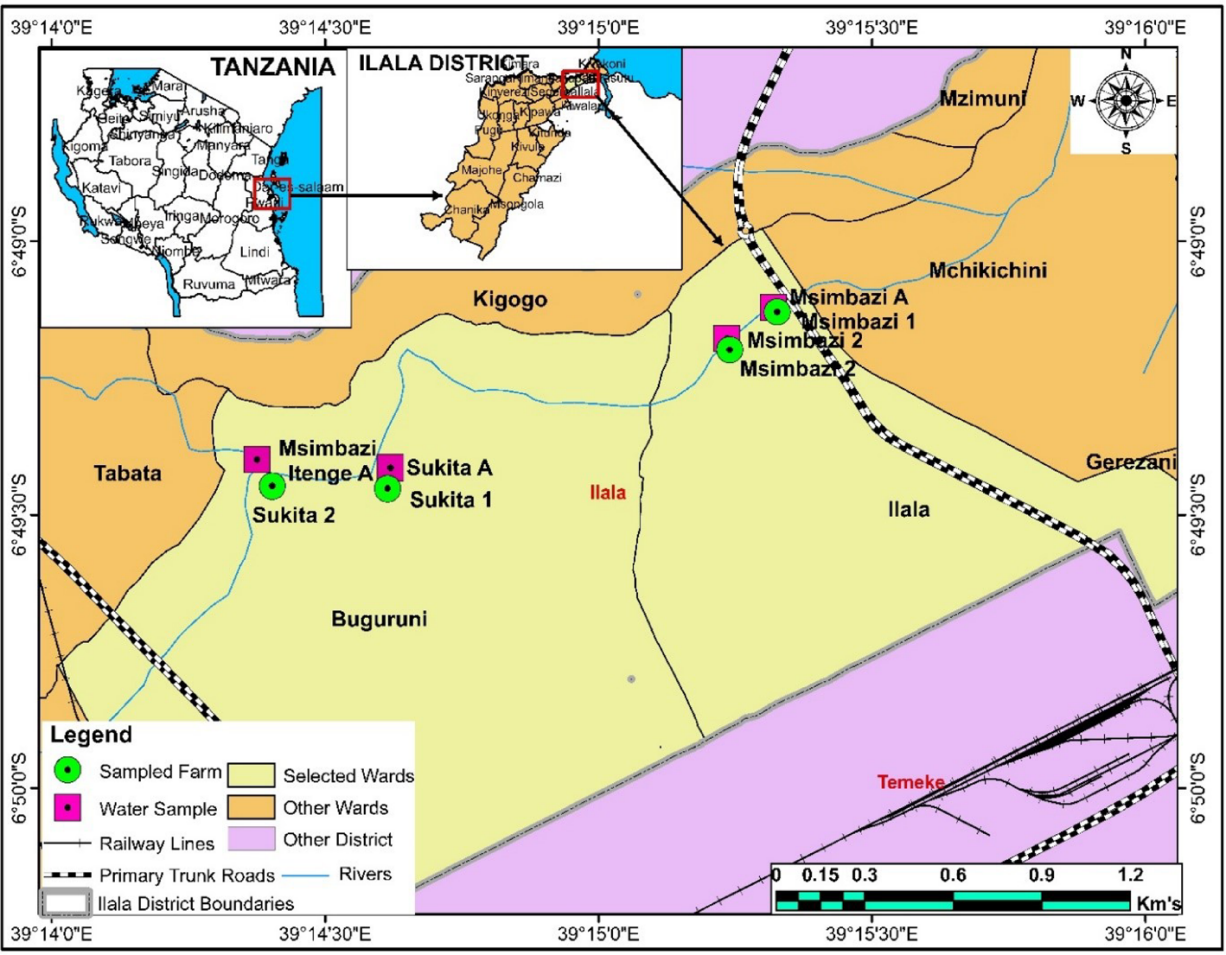
A map of Dar es Salaam city indicating water and soil sampling points in the Msimbazi River and adjacent Vegetable gardens

The Msimbazi basin, connecting three city municipalities of the Dar es Salaam region (Ubungo, Ilala, and Kinondoni), is a crucial area for vegetable cultivation. Despite being one of Tanzania's most densely populated regions, it serves as an important agricultural hub, with farmers practising year-round irrigation using water from the Msimbazi River [62]. The basin significantly distributes most cultivated vegetables to various locations in Dar es Salaam, primarily in nearby markets [126]. The Msimbazi River, nearly 45.25 km long and situated within a basin of 271 km^2^, originates from the Pwani region in the Kisarawe highlands (Pugu Forest) and discharges into the Indian Ocean around the Selander Bridge (Ilala-Dar es Salaam) [63]. Despite its vital role in agriculture, the basin is severely threatened by various sources of waste, including household and industrial waste. This threat is urgent and could potentially alter the biological and physicochemical properties of the basin and river water [59]. Water sampling points (-6°49′7.233", 39°15′19.1664") were chosen near the respective vegetable gardens (-6°49′11.91",39°15′14.3886"") as indicated on the map Fig. 1.

### Sampling

#### Water sampling

Sixty-four (64) water samples were collected in July 2023 when water from the Msimbazi River was used to irrigate adjacent vegetable gardens. At the Sukita sampling site, water samples were gathered from two main sub-sampling points, each at least 100 m apart. Sixteen (16) water samples were collected from each sub-sampling point, totalling thirty-two (32) from the Sukita sampling site. The same protocol was applied at the Msimbazi sampling site, where sixteen (16) water samples were also collected from both of the two (2) main sub-sampling points, resulting in thirty-two (32) water samples from this site. Before sample collection, the physicochemical water parameters, namely pH, temperature, dissolved oxygen (DO), total dissolved solids (TDS), and water conductivity (EC) at each sampling site were recorded on-site using a HI-9829 multi-parameter (Hanna Instruments, Italy) as described in Table 1.

**Table 1:** Physicochemical water parameters from the Msimbazi River

Parameter	Sukita Site	Msimbazi Site	Unit
pH	7.5	7.7	–
Total dissolved solids	425	485	ppm
Electrical conductivity	855	930	µs/m
Temperature	33	34	°C

Both in-line (on-site) and in-lab filtration methods were employed in this study, as previously demonstrated by Yuan et al. [149]. During sampling, three replicate water samples (approximately 5 L each) were directly procured from the Msimbazi River using a clean metallic bucket (15 L) and filtered into another clean metallic bucket of similar volume using a metallic sieve (5 mm pore size) to obtain a composite water sample containing MPs with a size of less than 5 mm. One (1) L of the filtrate was drawn into a 1.5 L glass bottle, covered with a metallic lid, placed in a cool box containing ice packs, and transported to the laboratory for further analysis according to the recommended methods and protocols by APHA [6].

#### Soil sampling

The vegetable gardens designated for soil sampling were identified as those adjacent to the Msimbazi River, which relies on water from the river for irrigation. Three replicates from a single point per garden were collected and mixed to form one composite sample; thus, sixteen (16) samples were gathered from two (2) gardens in Sukita and Msimbazi, respectively, totalling 32 composite samples per site. The four vegetable gardens, two (2) from each site, were located at least 100 m apart. Furthermore, at each garden, sixteen (16) sub-quadrats (0.32 × 0.32 m) were randomly selected and marked as points for collecting soil samples [22]. The soil cores (0–20 cm) were obtained from each sub-quadrat using a trowel and mixed to create one composite sample per field site, with 500 g collected per sample [1, 20, 85]. All samples were sealed in clean glass bottles with metal caps for further laboratory analysis [54].

### Isolation of microplastics from water samples

#### Sample pre-treatment for isolating microplastics from river water

For successive filtration and ease of observation of MPs, water samples were allowed to decant for about twelve (12) hours at room temperature (27 °C). Following decantation, the clear transparent part of the water was filtered under a vacuum filtration system using a membrane filter with a pore size of 0.45 µm [56, 117]. The filter paper was then stored in a clean glass petri dish, ready to be examined for quantification of MPs under a stereomicroscope [81]. The remaining unfiltered sediment part of the water samples was dried in an oven at 60 °C before being subjected to the oxidation of organic matter.

#### Organic matter removal from water sediments

The oven-dried organic matter from water samples (unfiltered water) was removed by an oxidation process based on Fenton's reagent as described by Hurley et al. [48]. Hydrogen peroxide (H_2_O_2_) (30%) was employed for the oxidation process, catalysed by the addition of ferrous sulphate (FeSO_4_.5H_2_O) (0.02 M). The pH of the reaction mixture was adjusted to 3.0, as outlined by Neyens et al. [90]. The temperature was constantly maintained below 40 °C by placing the beaker containing the reaction mixture on ice cubes to prevent the loss of H_2_O_2_ before ending the reaction. The end of the reaction was indicated by a change in colour and the disappearance of air bubbles (oxygen and carbon dioxide gas) following the addition of an excess of H_2_O_2_ and FeSO_4_.5H_2_O [117]. Ultimately, the reaction mixture was oven-dried to reduce excess water before isolating MPs.

#### Density separation technique for isolating microplastics from water sediments

A density separation technique utilising zinc chloride (ZnCl_2_) salt, with a density (ρ) of 1.8, was adopted to isolate MPs from the oven-dried oxidised water sediment samples, as described by Coppock et al. [24]**.** An aqueous solution of ZnCl_2_ was added to a beaker containing the oven-dried and oxidised sediment sample at a ratio of 3:1 (volume-to-volume), covered with aluminium foil, and placed on a magnetic stirrer for 30–45 min at 800 revolutions per minute (rpm). The reaction mixture was allowed to settle (decant) for 48 h to permit heavy and non-plastic materials to settle, while lower-density particles (plastic materials) floated in the upper aqueous layer, as described by Neyens et al. [43]. The aqueous part was filtered on a vacuum filtration unit using a 0.45 µm membrane filter and stored in a clean glass petri dish [56, 117]. The extraction of MPs using ZnCl_2_ was repeated three times on the same sample to isolate a sufficient quantity.

### Pre-treatment, oxidation, and isolation of microplastics from vegetable garden soils

**Fig. 2:**
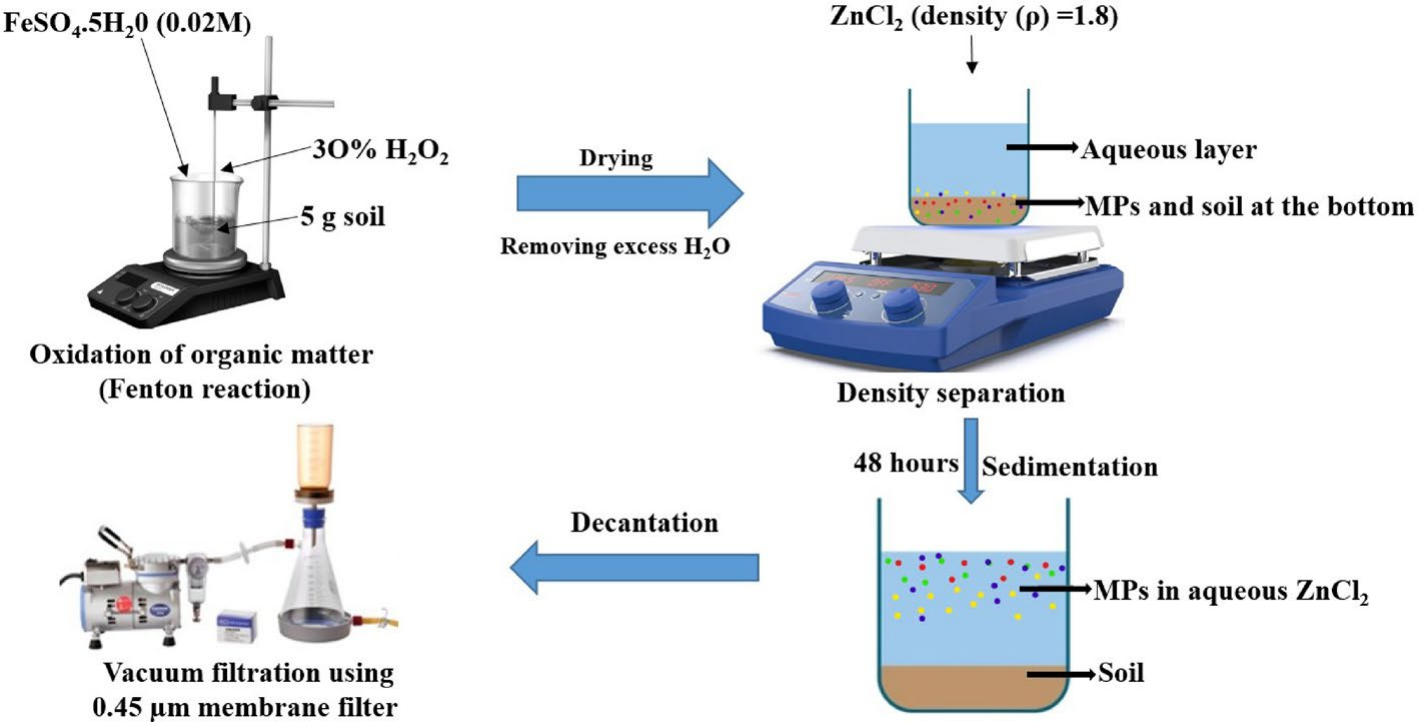
Isolation of microplastics (MPs) from soil samples by the density separation method

The soil samples from vegetable gardens were dried in an oven at 60 °C until completely dry, then allowed to cool at room temperature (27 °C) in a laminar flow before being sieved with a 5 mm metallic sieve to remove larger objects. A 5 g portion of the oven-dried soil sample, sized less than 5 mm, was placed into a clean 100 mL beaker for the oxidation process to eliminate organic matter before undergoing density separation. The oxidation of organic matter from the soil samples employed Fenton's reagent and adhered to the same protocol as for water sediment. The oxidation of organic matter from soil samples, density separation of oxidised soil samples, and sedimentation for obtaining MPs in the aqueous layer, as well as the filtration of MPs from the aqueous layer, were conducted thoroughly and comprehensively, as shown in Fig. 2.

### Enumeration of microplastics isolated from water and soil samples

Following the laboratory vacuum filtration, the particles of MPs retained on the membrane filters were observed and counted using a stereomicroscope (OPTICA Microscope ITALY, WF 20X/10) [105, 155]. The observation of MPs was facilitated by moistening the dried membrane filters containing the MPs with 70% ethanol and counting with the help of a thin-tipped tweezer. The isolated and identified particles of MPs were placed on Fourier transform infrared spectroscopy (FTIR) for reliable confirmation [25, 127].

### Characterization of microplastics by Fourier transform infrared spectroscopy

ATR-FTIR-Spectrophotometer-Shimadzu IRSprit coupled with single-reflection attenuated reflection transform infrared spectroscopy (QATR-S) was employed to identify the polymers of MPs. The wavelength selected was between 400 and 4000 cm^−1^ with 20 scans at the sensitivity of one (1) at a resolution of 4 cm^−1^ for processing spectra data as described by Circelli et al. [23]. Before the analysis, the QATR-S crystal was cleaned with smooth tissue soaked in absolute ethanol (99.99%) and dried; an anvil was then allowed to contact the QATR-S crystal to run the background for 2–4 min [88]. Following the background run, one drop of absolute ethanol (99.99%) was placed on the QATR-S crystal, which aided in securing the MPs (> 567 µm) on the crystal. The MPs were observed under a microscope and isolated from ethanol-wet filter paper using a thin-tipped tweezer [12, 137]. Subsequently, the anvil was permitted to contact the MPs added to the QATR-S crystal for measurements. The system reference library and open-source tools, such as Open Specy, were highlighted for their role in comparing the resultant spectra [28, 103].

### Quality control and assurance

To avoid contamination of MPs, the study utilised non-plastic materials, namely glass bottles with metallic lids, for water and soil sampling, as previously demonstrated by Crossman et al. [26]. Latex gloves and cotton laboratory cloths were preferred during the laboratory work to avoid the introduction of foreign MPs to the samples [77]. Furthermore, most of the laboratory work was performed in a laminar flow cabinet and a controlled, closed room (for experiments that were challenging to conduct in a laminar flow cabinet, such as vacuum filtration) [122]. Lastly, chemicals and reagents, including distilled water (blank) used in the laboratory analysis, were filtered through 0.45 µm membrane filters and examined under a stereomicroscope (OPTICA Microscope ITALY, WF 20X/10) to ensure they were free of MPs.

### Statistical analysis

The mean values and standard deviation of water and soil samples were calculated and reported as mean ± standard deviation [56]. Thirty-two (32) water and soil samples were treated separately based on two sampling locations along the Msimbazi River. Normality (Shapiro–Wilk) and homogeneity of variance tests were used to analyse the test data; hence, a two-sample t-test was used to assess the mean significance of Msimbazi and Sukita samples and recorded as a P-value at a 95% confidence level. P value < 0.05 was treated as the mean statistical significance [80]. Principal component analysis and hierarchical cluster analysis were also performed to identify the distribution and prevalence of six major polymers of MPs in water and soil samples from the Msimbazi and Sukita sampling sites, respectively [89]. All statistical analysis and graph plotting were performed using Origin Pro 2024 software [18].

## Results and discussion

### Images of microplastics isolated from water and soil

**Fig. 3:**
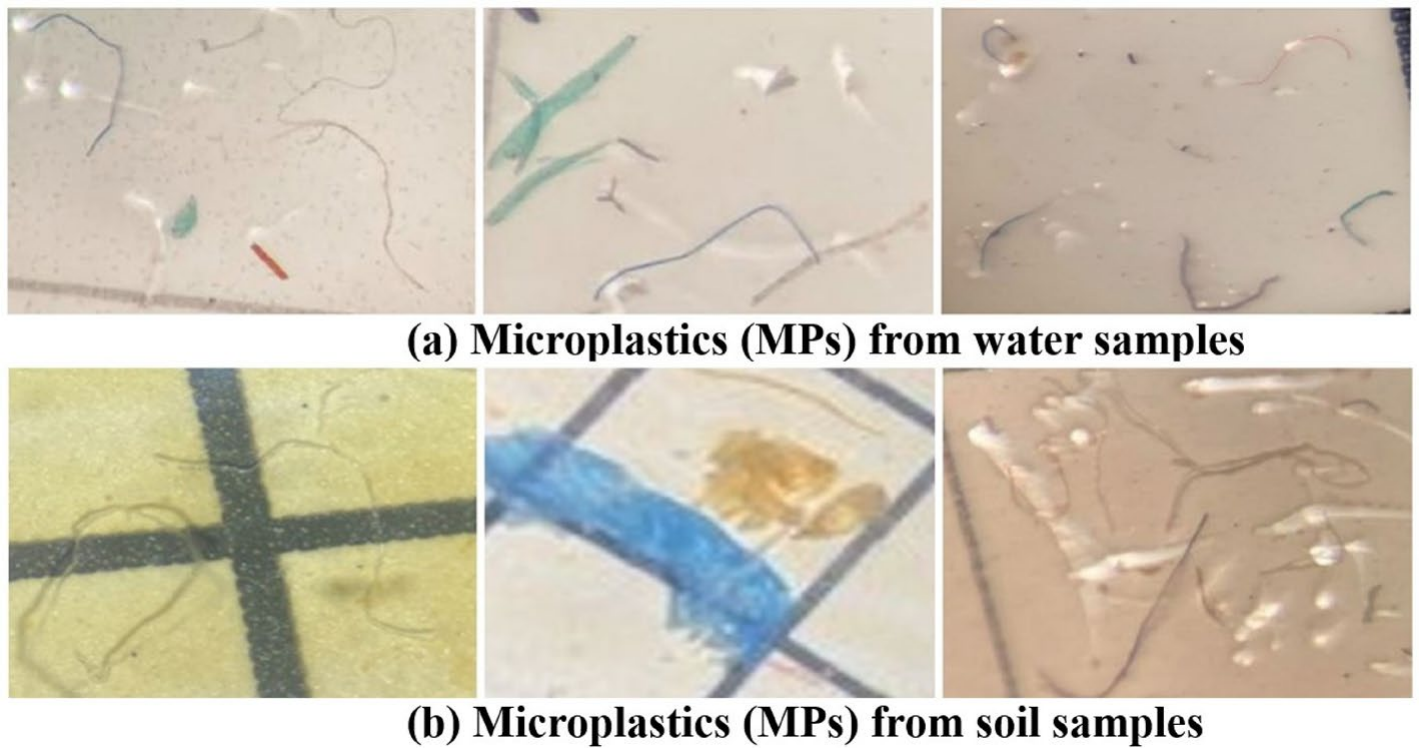
Images of Microplastics (MPs), **a** from water samples, and **b** from soil samples under × 20 magnification factor of a stereomicroscope

Microplastics from water and vegetable garden soils were pre-confirmed by observing under a stereomicroscope (OPTICA Microscope ITALY, WF 20X/10) [130]. Figure 3a shows the image of the MPs from water samples; Fig. 3b, represents the image of MPs from the vegetable gardens' soil.

### Abundance of microplastics in water samples from the Msimbazi river

**Fig. 4:**
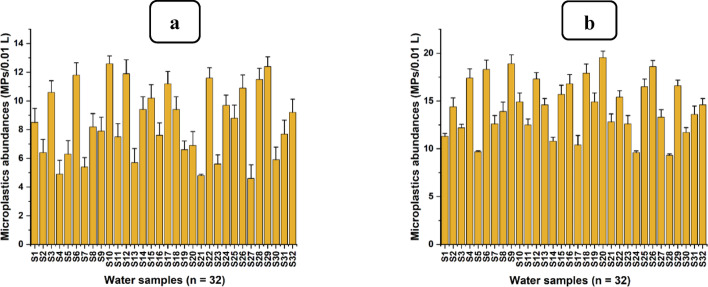
Concentration of microplastics from water samples, **a** Sukita site (upstream), and **b** Msimbazi site (downstream) along the Msimbazi River

The current study assessed the concentrations of MPs in water collected from Sukita and Msimbazi sites along the Msimbazi River. All sixty-four (64) samples that were analysed from the Sukita (n = 32) and Msimbazi (n = 32) sample-collection sites contained significant levels of MPs as indicated in Fig. 4.

The abundance of MPs across all sites ranges from 460 (upstream) to 1953 (downstream) MPs per litre (L). Statistical analysis using the normality test (Shapiro–Wilk) revealed significant differences among the thirty-two (32) samples from the Msimbazi site (downstream), with a p-value of 0.49, indicating they were normally distributed. At a 95% confidence level and degrees of freedom (df = 31), the mean value for the concentration in downstream water samples was 14.32 ± 2.917 MPs per 10 mL, with concentrations of 13.280 ± 2.917 and 15.384 ± 2.917 MPs per 10 mL representing the lowest and highest values, respectively. Similarly, results from the Sukita sampling site (upstream) were normally distributed (Shapiro–Wilk) with a p-value of 0.129. Moreover, at a 95% confidence level and degrees of freedom (df = 31), mean concentration in the upstream site was 8.491 ± 2.475 MPs per 10 mL, with minimum and maximum concentrations of 7.598 ± 2.475 and 9.383 ± 2.475 MP_S_ per 10 mL, respectively. A two-sample t-test, assuming equal variances between the water sampling sites (df = 62), demonstrated a significant difference (p-value < 0.001) between the samples collected from the upstream and downstream sites. Consequently, water samples collected downstream of the Msimbazi River (Msimbazi site) contained a comparatively significant amount of MPs compared to those from upstream (Sukita site).

Results from the current study indicate that the quantity of MPs in water samples increases from upstream (Sukita site) to downstream (Msimbazi site); this was also observed by Liu et al. [75]. Various factors, including the increase in the population downstream of the Msimbazi River, which escalates human activities, result in the disposal of a larger amount of plastic waste, which degrades into MPs [45, 46, 145]. Additionally, the area between upstream and downstream is vulnerable to flooding. Consequently, residents often deposit significant quantities of plastic waste along the riverbanks as makeshift barriers to mitigate flooding, serving as secondary sources of MPs into the Msimbazi River, as depicted by Liro et al. [74]. Therefore, mean differences in the concentration of MPs at the sampling sites of the Msimbazi River, like in many other rivers, depend on the distance from upstream to downstream approaching the ocean [35, 125]. This is due to riparian hydrodynamic parameters (tides and waves) influencing the dispersion and suspension of MPs [111].

The results generally show that the Msimbazi River is contaminated with MPs, mainly due to industrial effluents and plastic waste dumped into the river or its shorelines [7, 16, 128]. The lack of well-organized solid waste collection services (poor community involvement) in the areas adjacent to the Msimbazi River makes residents opt to dump solid waste, particularly plastics, into the River, which is easy, at no cost, and fast, compared to waiting for waste collectors, which is costly and takes time [33, 65, 111]. Based on the current study's results, water from the Msimbazi River contains a sufficient amount of MPs and is still used for surface irrigation of vegetable gardens adjacent to the river. Therefore, water from the Msimbazi River serves as a substantial source of MPs for the soil of vegetable gardens and the vegetables themselves, as surface irrigation was identified as the primary source of MPs for the soil and vegetables by Yu et al. [148].

The current concentration of 460–1953 MPs per litre (L) in water from the Msimbazi River in Dar es Salaam, Tanzania, is comparable to other studies based on similar protocols (Table 2). However, this comparison is imperfect, as various studies have utilised different protocols for sample collection and analytical experiments. For instance, the selection of salt for density separation, the density of the salt used, the pore size of the filter paper, and the magnification factor of the microscope for observing and counting MPs [118]. Nevertheless, our current study represents a more substantial quantity of MPs from river water than similar studies, except for those conducted on the eastern coast of China by Nkosi et al. [92].

**Table 2:** Comparison of the number of microplastics (MPs) from surface water of global rivers and agricultural soils relative to the Msimbazi River and its adjacent vegetable gardens

Location	Country	Concentration	Polymer type	Reference
Awano River	Japan	132 ± 15 MPs/L	PP, PE, Vinylon	Kabir et al. [58]
Buriganga River	Bangladesh	250–117 MPs/L	PET, HDPE, Nylon, CA, and ABS	Hague et al. [41]
Yongjiang River	Nanning City (South China)	550–770 MPs/L	PET, PP, PE, Nylon, PS	Zhang et al. [154]
Fenghua and Ningbo Rivers	The eastern coast of China	300–4000 MPs/L	PP, LDPE, HDPE, acrynitrile styrene	Xu et al. [144]
Werrible River	Australia	22 ± 11 MPs/L	PET, PP, PE, PA, PS,	Samandra et al. [123]
Crocodile River	Nelspruit City (South Africa)	625–1058 MPs/L	Fibres and fragments	Nkosi et al. [92]
Msimbazi River	Dar es Salam city (Tanzania)	460–1953 MPs/L	PET, LDPE, HDPE, PS, and PP	This study
Lahore city	Pakistan	4483 ± 2315 MPs/Kg	Fragments, sheets, beads, and foam	Rafique et al. [108]
Yangtze River	Shanghai (China)	4.94–252.71MPs/Kg	PP, PE, Nylon, PA, PS	Cao et al. [14]
Chai River	China	7100–42960 MPs/Kg	Fibers, strings, film, fragments	Zhang et al. [152]
Vegetable plots	China (Wuhan)	4.3 × 10^4^- 6.2 × 10^5^ MPs/Kg	PE, PP, PS, PA, PVC	Zhou et al. [157]
Urban-roads, landfill-associated, and woodland sites	Southern England (UK)	12,200–17300 MPs/Kg	PE, PP, and PS	Billings et al. [12]
Vegetable gardens	Dar es Salaam—Tanzania	20,100–44300 MPs/Kg	PP, LDPE, PS, and PET	This Study

PP, (polypropylene); PE, (polyethylene); PET, (polyethylene terephthalate); HDPE, (high-density polyethylene); CA, (cellulose acetate); ABS, (acrylonitrile butadiene); AS, (acrynitrile styrene); PS, (polystyrene); LDPE, (lower-density polyethylene); PA, (polyamide); PVC, (polyvinyl chloride)

### Abundance of microplastics in soils of vegetable gardens near the Msimbazi River

**Fig. 5:**
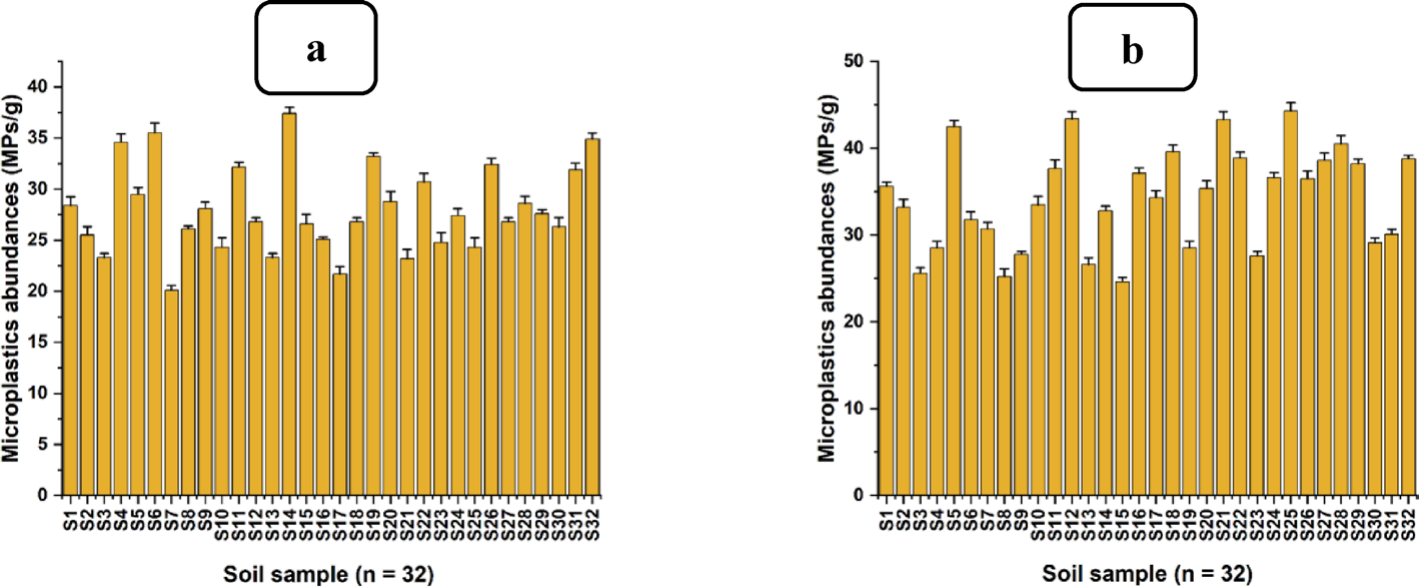
Microplastics (MPs) concentrations isolated from the soil of **a** Sukita vegetable gardens site, and **b** Msimbazi vegetable gardens site

The present study uncovered a significant environmental issue: the MPs contamination in soils from Sukita and Msimbazi vegetable gardens, which are adjacent to the Msimbazi River, as depicted in Fig. 5.

Soil samples collected from Sukita vegetable gardens, as shown in Fig. 5a, contained the lowest amount of MPs (20.10 MPs per g). In contrast, the highest amount of MPs was found in soil samples collected from the Msimbazi vegetable gardens (44.30 MPs per g), as indicated in Fig. 5b. The normality test (Shapiro–Wilk) was conducted and indicated significant differences among the soil samples (n = 32) collected from Sukita vegetable gardens, with a p-value of 0.97. Moreover, at a 95% confidence level and with 31 degrees of freedom (df = 31), the mean concentration was 28.01 ± 4.25 MPs per g, with 20.10 ± 4.25 and 37.40 ± 4.25 MPs per g being the lowest and highest concentrations, respectively. Similarly, there were significant differences following the normality test (Shapiro–Wilk) with a p-value of 0.96 among the soil samples (n = 32) collected from the Msimbazi vegetable gardens, thus confirming that they were normally distributed. At a 95% confidence level and with 31 degrees of freedom (df = 31), the average MPs concentration from the soil of Msimbazi vegetable gardens was 34.28 ± 5.79 MPs per g, with the least and highest abundances of 24.6 ± 5.59 and 44.3 ± 5.79 MPs per g, respectively. A two-sample t-test assuming equal variances between the soil samples from Sukita and Msimbazi vegetable gardening sites indicated significant mean statistical differences with a p-value of less than 0.0001 between the two sites (df = 62). Thus, soil samples collected from Msimbazi vegetable gardens contained a relatively significant amount of MPs compared to those from Sukita vegetable gardens.

The results from the current study indicate that soil samples from Msimbazi vegetable gardens, as depicted in Fig. 5b, situated downstream of the Msimbazi River, had a high concentration of MPs compared to soil from Sukita vegetable gardens as shown in Fig. 5a. This is likely due to several factors, including the significant amount of MPs in irrigation water downstream of the river, which can contribute to a high load of MPs in Msimbazi garden soil [100]. Furthermore, numerous unauthorized solid waste dumping sites, including plastics, were identified near Msimbazi vegetable gardens and river shorelines, which contribute to the release of MPs into the soil of nearby vegetable gardens and in the Msimbazi River [9, 37]. Moreover, the area surrounding the Msimbazi vegetable gardens is densely populated, and the vegetable gardens are located closer to houses, hence there is greater tendency of dumping plastic waste into the gardens as compared to the Sukita vegetable gardens, which are situated in an industrial area; therefore, the population is lower, and houses are not near the gardens [33, 95].

However, both vegetable gardening sites are situated in the Msimbazi basin area, which is highly prone to flooding. Consequently, Msimbazi vegetable gardens site downstream of the Msimbazi River is more susceptible to flooding than the upstream vegetable gardens at the Sukita site. Flooding introduces numerous contaminants, including muddy water containing MPs and macroplastics that potentially contribute significantly to MPs in the soils of Msimbazi vegetable gardens [135, 140]. Also, human activities downstream of the Msimbazi River basin exacerbate the contamination of agricultural soil with MPs, primarily through the illegal dumping of solid waste, particularly plastic waste, whose degradation leads to the generation of MPs [108, 156]. Given that the Msimbazi vegetable gardens are located near urban roads, the notable quantity of MPs in their soil may be attributed to tire wear particles compared to soil from Sukita vegetable gardens, which are situated on non-urban roads. A similar incidence was observed in the study conducted by Yuling et al. [18]. Regardless of the differing methodological approaches utilised for isolating MPs from soil samples, the levels of MPs reported in the present study are comparable with similar studies, as illustrated in Table 2 [14, 152, 157].

Our study has revealed a significant occurrence of MPs in water and soil samples from the Msimbazi River and its adjacent vegetable gardens. This discovery underscores the various migration pathways of MPs from river water to the soil of vegetable gardens via irrigation. We specifically examined the direct transfer of MPs through surface irrigation, where MPs suspended in water are deposited into the soil during irrigation [40, 47, 147]. However, it's essential to note that other irrigation pathways also contribute to the migration of MPs, including subsurface infiltration [94, 129], sprinkler/overhead irrigation [2, 84], and drip irrigation [32]. Hence, practically all forms of irrigation pathways that transport water contaminated with MPs, which reach agricultural fields, facilitate the transfer of MPs to those fields [115]. Moreover, other environmental pathways might also contribute to conveying MPs to farming fields, including atmospheric deposition of MPs [143], flooding [10, 102], sediment transport during irrigation runoff [38, 67], and the use of organic fertilizers and animal manure [71, 114, 153]. This highlights the need for further research to comprehend the scope of this issue fully.

Thus, it is not just about MPs contamination in irrigation water and agricultural soils; their occurrence presents a significant threat to public health and the environment. Numerous studies have highlighted the detrimental effects of MPs, including their potential to act as carriers for pathogenic pathogens and genes that confer resistance [51, 57, 106, 134]. This underscores the urgency of addressing this issue. Ultimately, vegetables grown in the Sukita and Msimbazi vegetable gardens may also be contaminated with MPs. This is because the irrigation water and the soil in which they are grown are polluted with substantial amounts of MPs; a similar scenario was also observed by Nuno et al. [13].

### Polymer composition of isolated microplastics

**Fig. 6:**
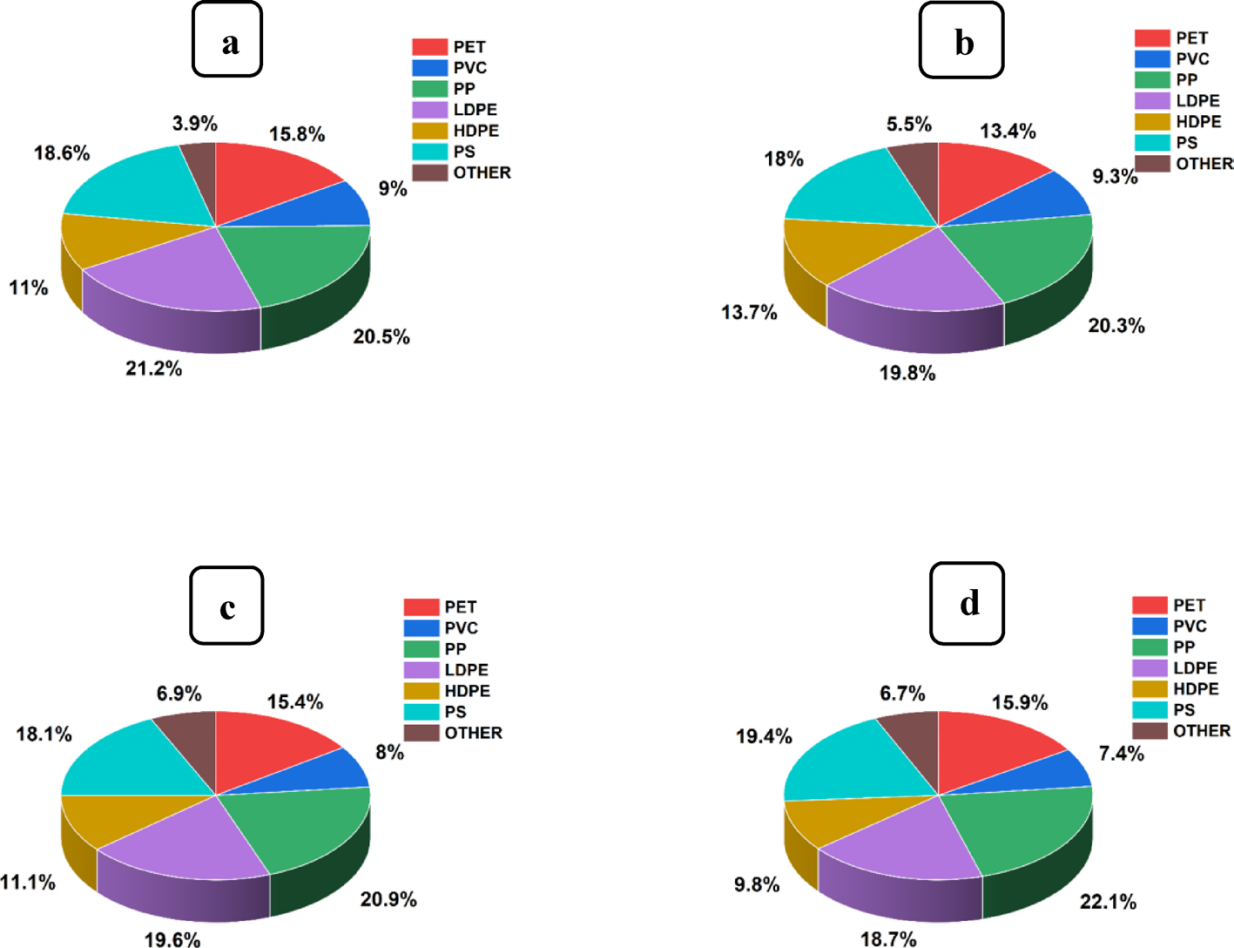
Microplastics (MPs) polymer type and their composition of the Msimbazi River water samples **a** Sukita site (upstream) and **b** Msimbazi site (downstream) and soil samples **c** Sukita vegetable gardens and **d** Msimbazi vegetable gardens

Utilising the FTIR technique, the present research revealed more than six (6) types of polymers of MPs in soils from vegetable gardens and irrigation water samples in the Msimbazi basin, with their FTIR spectra (Supplementary Fig. S1). The most prevalent of these was polypropylene (PP), a widely used plastic polymer [142], as illustrated in Fig. 6, and Table 3.

**Table 3:** Percentage composition of plastic polymers of water and soil samples

Percentage composition of MPs by polymer	Water samples (%)		Soil samples (%)	
	Msimbazi site	Sukita site	Msimbazi site	Sukita site
Polyethylene terephthalate (PET)	13.40	15.80	15.90	15.40
Polyvinyl chloride (PVC)	9.30	9.00	7.40	8.00
Polypropylene (PP)	20.30	20.50	22.10	20.90
Low-density polyethylene (LDPE)	19.80	21.20	18.70	19.60
High-density polyethylene (HDPE)	13.70	11.00	9.80	11.10
Polystyrene (PS)	18.00	18.60	19.40	18.10
Other	5.50	3.90	6.70	6.90

The percentages of PP polymers in the water samples from the Sukita and Msimbazi sampling points were 20.5% and 20.3%, respectively. However, soil samples contained a relatively high percentage of PP polymers, with 22.1% from soil samples of Msimbazi vegetable gardens and 20.9% from Sukita vegetable gardens.

Lower-density polyethylene (LDPE) was the second most abundant plastic polymer in water and soil samples, likely influenced by the widespread use of thin plastic carrier bags [132]. The LDPE MPs were most dominant in water samples compared to soil samples, which were quantified as 21.2% and 19.8% in water samples from Sukita and Msimbazi sampling sites, respectively. Meanwhile, in soil samples, LDPE MPs were identified at 19.6% and 18.7% in the Sukita and Msimbazi vegetable garden soils, respectively, suggesting a lesser but still substantial presence in the soil. Polystyrene (PS) was also quantified in high proportions, coming second to PP and LDPE, and was predominantly found in vegetable garden soil samples compared to water samples. Notably, soil samples from the Sukita gardens contained 18.1% PS, while those from the Msimbazi gardens had 19.4% PS. Similarly, water samples from Sukita site had 18.6% PS, whereas those from Msimbazi site had 18% PS. Despite PET plastic polymers being the most recycled plastic material in Dar es Salaam, Tanzania [61, 146], a substantial quantity of its polymers of MPs was detected in the current study, with 15.8% and 13.4% obtained from water samples at the Sukita and Msimbazi sites, respectively, along the Msimbazi River. In contrast, 15.4% and 15.9% were determined from soil samples of Sukita and Msimbazi vegetable gardens, respectively, adjacent to the Msimbazi River. Therefore, the highest enumeration of PP, LDPE, PS, and PET in the soil and water samples in the present study highlights the urgent need for further research and potential mitigation strategies.

Unlike other plastic polymers in the current study, HDPE and PVC were among the least identified plastic polymers in all water and soil samples. This could primarily be due to their reusability; thus, they are often removed from the environment by waste pickers for recycling and reusing [133]. Other MPs polymers identified in the water and soil samples included polyurethane (PUR), polyamide, polyesters, and fibre thermoplastics. Therefore, the presence of similar types of MPs polymer compositions in water from the Msimbazi river used for irrigation and soils of nearby vegetable gardens suggests a possible vertical distribution of MPs in vegetable garden soils, which is likely caused by irrigation water and appears to be the primary source of MPs [147].

Despite the prohibition on single-use PE plastic carrier bags and the introduction of reusable bags (PP) in the United Republic of Tanzania. The current study has demonstrated a high prevalence of LDPE and PP MPs in both soil and water samples. Moreover, an emerging current environmental concern is the widespread use of thin LDPE plastic bags as an alternative to the prohibited single-use PE plastic carrier bags. Thus, these findings underscore the significant prevalence of MPs polymers in the Msimbazi basin, highlighting the urgent need for further research and the development of potential mitigation strategies.

### Source identification

#### Principal component analysis

**Fig. 7:**
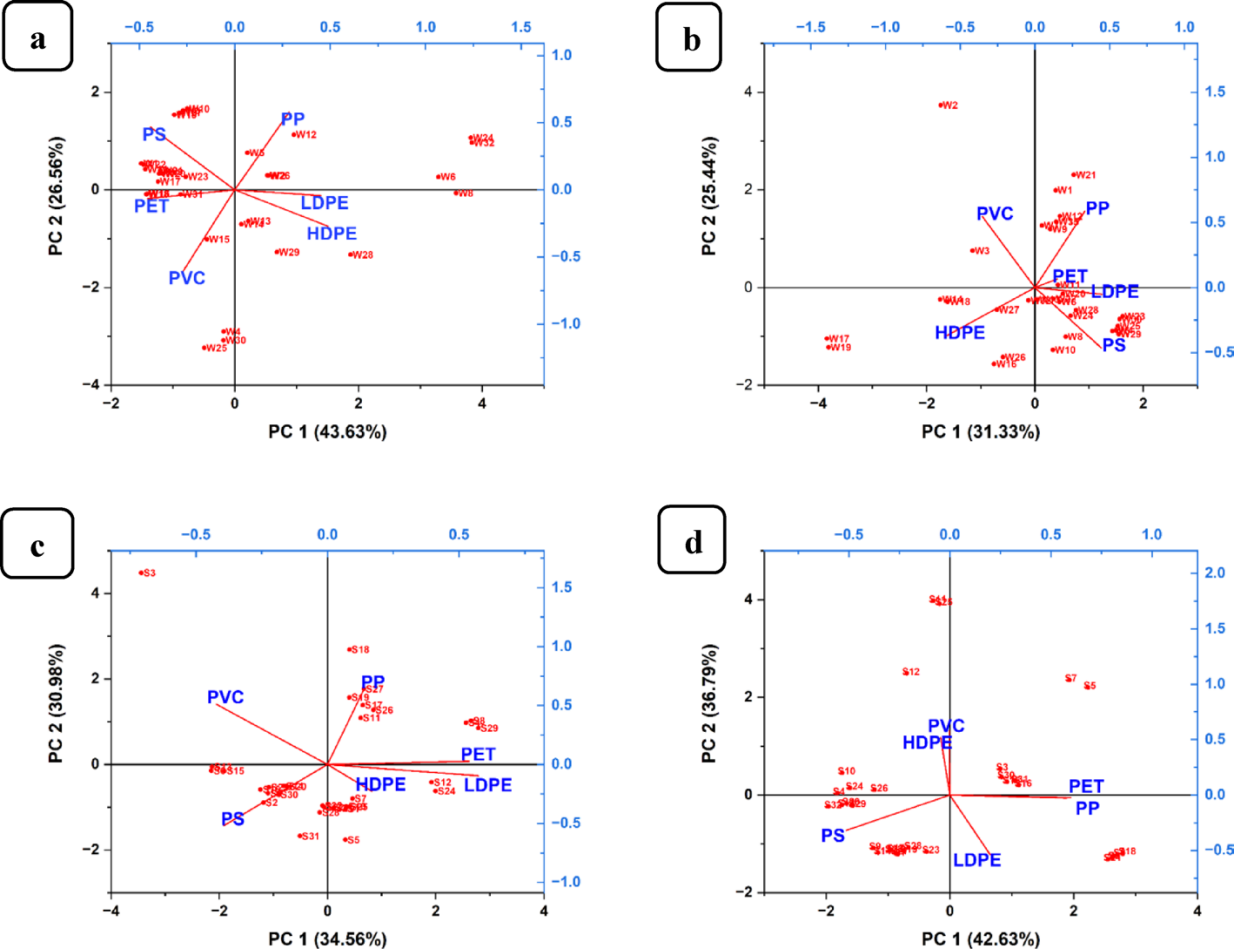
Principal component analysis for identification of sources of MPs polymers between water samples **a** Msimbazi site and **b** Msimbazi site, and as well as between soil samples **c** at Msimbazi site and **d** Sukita site, respectively

Principal component analysis was used to illustrate the distribution of polymers of MPs, i.e., HDPE, PP, LDPE, PET, PVC, and PS, between soil and water samples at their respective sample collection points along the Msimbazi River. Figure 7 (a-b) shows the MPs polymer distribution between water samples at Msimbazi sampling site (n = 32), Fig. 7a, and Sukita sampling site (n = 32), Fig. 7b.

The analysis shows polymers of MPs were well distributed across the Msimbazi water sampling site, as shown in Fig. 7a, with PC1 (43.63%) comprising (PET, LDPE, PVC, and HDPE), and PC2 (26.56%) including (PP and PS). This contrasts with the Sukita water sampling site indicated in Fig. 7b, where PC1 (31.33%) consisted of (HDPE, PS, and LDPE), and PC2 (25.44%) was dominated by (PVC and PP). Similarly, Fig. 7 (c-d) illustrates the distribution of MPs' polymers in soil samples from the Msimbazi vegetable gardens site (n = 32), as shown in Fig. 7c, and the Sukita vegetable gardens site (n = 32), shown in Fig. 7d. In Msimbazi garden soils, as observed in Fig. 7c, MPs polymers were less distributed, with PC1 (34.56%) for (PS, HDPE, and LDPE), and PC2 (30.98%) for (PVC, PP, and PET). In contrast to samples from Sukita garden soils, as described in Fig. 7d, the polymers of MPs were distributed with PC1 (42.63%) for (PS, LDPE, PET, and PP), and PC2 (36.79%) for (PVC and HDPE). According to the results of the current study, the primary sources of MPs in the soil were irrigation water and the disposal of household solid waste, such as plastic bags, household utensils, and PET bottles [99]. Likewise, the sources of MPs in the Msimbazi River include dumping of household solid waste and discharge of industrial wastewater effluents [109]. The implications of these findings are significant and highlight the urgent need to address pollution from MPs.

#### Hierarchical cluster analysis

**Fig. 8:**
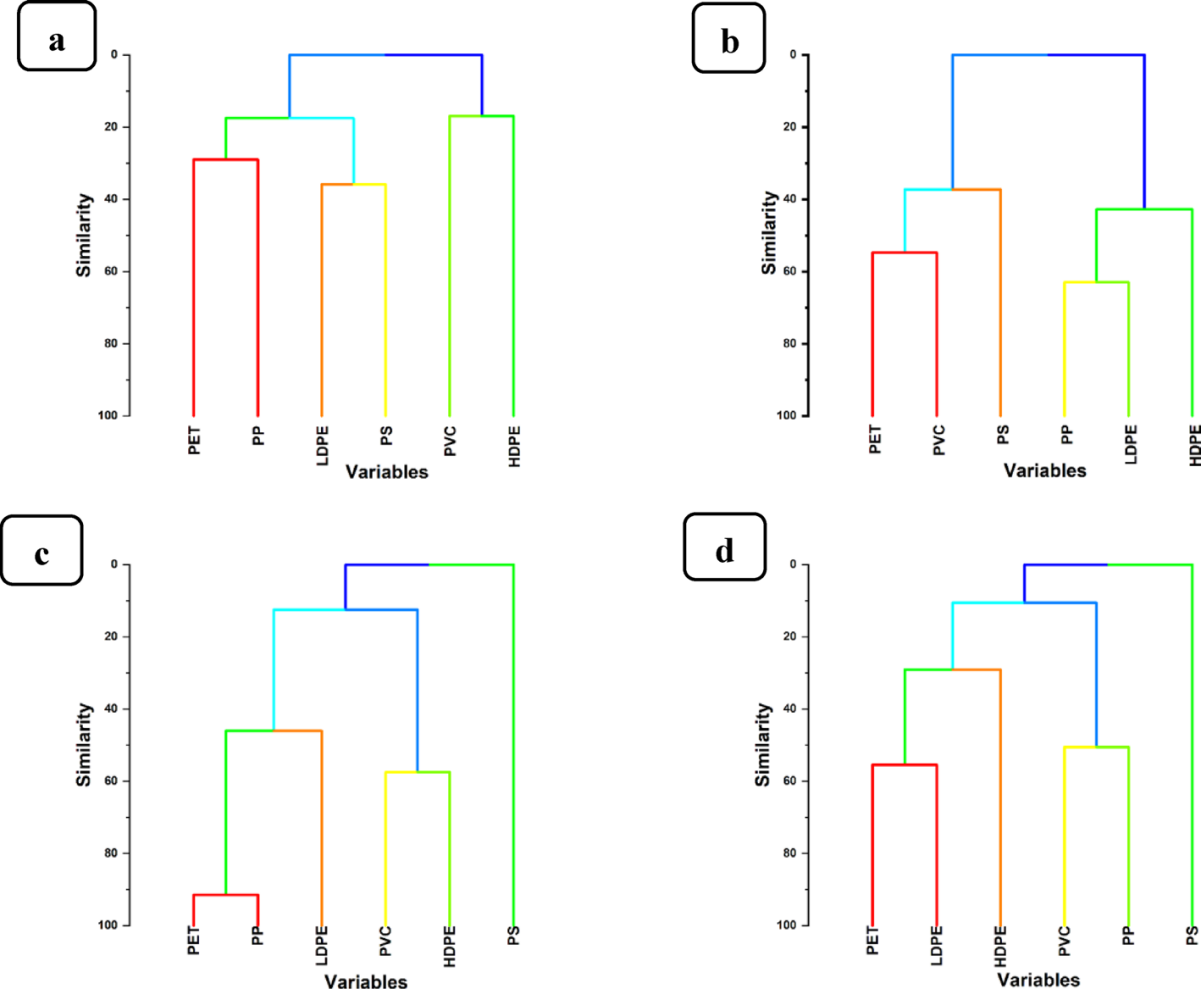
Hierarchical clustering dendrogram of prevalence of MPs polymers **a** water samples from Sukita site **b** waters samples from Msimbazi site **c** soil samples from Sukita vegetable gardens, and **d** soils from Msimbazi vegetable gardens

Results obtained from the hierarchical cluster analysis of each of the thirty-two (32) experimental samples of water from the Msimbazi River and soils from adjacent vegetable gardens, respectively, are presented in Fig. 8.

The thirty-two (32) samples, each obtained from river water collected at Sukita and Msimbazi sampling sites, as well as soils from Sukita and Msimbazi vegetable gardens, were analyzed to determine the most and least prevalent MPs polymers, including LDPE, PP, PVC, PET, PS, and HDPE. For example, water samples collected from Sukita sampling site (upstream) of the Msimbazi River (Fig. 8a) contained PP as the most prevalent MPs polymer and HDPE as the least. At the same time, water samples from Msimbazi site (downstream) of the River (Fig. 8b) had LDPE as the most prevalent MP polymer and HDPE as the least. However, for soil samples collected from the Sukita vegetable garden site, as shown in Fig. 8c, PP was the most observed polymers of MPs, and PS was the least. While at the Msimbazi garden site, Fig. 8d, PET was the most prevalent polymer and PS the least prevalent. Thus, as also observed by Nayrac et al. [89], PP and LDPE are the most abundant MPs polymers found in the water of the Msimbazi River, as well as in the soils of vegetable gardens adjacent to the Msimbazi River.

### Limitations of the study

The limitations observed during the conduct of the current study include the quantification of MPs from soil and water samples, which is likely to be underestimated due to several factors, including the recovery of MPs after extraction. At which three repeated extractions of the MPs extraction process per sample, using ZnCl_2_ as recommended by previous studies [44], were not sufficient. We recommend increasing the number of extractions per sample in further studies to isolate the maximum quantity of MPs.

In this study, enumeration of MPs was based on visual observation using a stereomicroscope; however, there is a greater possibility of human error, as some of the MPs were very small and closely packed together, which makes it difficult to count [151]. Moreover, the current study employed a density separation method using ZnCl_2_ salt, which is capable of dissolving with a greater density (more than 1.6 g/cm^−3^). This method is effective in isolating almost all types of MPs, including MPs of high density, such as PVC (1.16–1.58 g/cm^−3^) [79]. Thus, the experiments were limited to a small number of samples (n = 32) per sampling site due to the high procurement cost of ZnCl_2_ and related consumables.

Furthermore, a prominent number of MPs were observed per membrane filter, resulting in only a few MPs being considered for FT-IR analysis. This was due to the high cost and time-consuming nature of conducting FT-IR analysis on all observed MPs from each sample. Lastly, during FTIR analysis for polymer identification of MPs, µFTIR was not employed. Instead, the study relied on relatively larger observed MPs (0.567-5 mm), aided by an eyepiece and a stereomicroscope, while MPs smaller than 0.567 mm remained unidentified [12, 99].

## Conclusion

The current study reveals that water from the Msimbazi River, a crucial source for irrigating vegetables, contains a significant amount of MPs up to 1953 MPs/L. The study also reveals that soil from vegetable gardens adjacent to the Msimbazi River is polluted, with a maximum of 44.3 MPs/g. In 2019, the government of the United Republic of Tanzania introduced a law that bans single-use plastic carrier bags while promoting the use of reusable PP bags. Still, the FT-IR analysis in our study identified the highest percentages of PP and LDPE MPs polymers, along with PET and PS in moderate amounts. Thus, plastic waste management requires urgent actions, particularly in educating the public about effective plastic waste management strategies, such as recycling, reusing, reducing the use of single-use plastics, and sorting at the source. Additionally, governments should implement the 'polluter pays principle', which holds producers accountable for the end-of-life costs of their plastic products, and 'extended producer responsibility policies', which require producers to retrieve and recycle their products. Further research is needed to understand the sources, distribution, and impacts of MPs on human health and ecosystem integrity.

## Supplementary Information

Below is the link to the electronic supplementary material.

Supplementary Material 1

## Data Availability

The datasets/information used and generated in the current study will be available from the corresponding authors upon reasonable request.
